# Perception of Physical Fitness and Exercise Self-Efficacy and Its Contribution to the Relationship between Body Dissatisfaction and Physical Fitness in Female Minority Children

**DOI:** 10.3390/ijerph15061187

**Published:** 2018-06-06

**Authors:** Emily W. Flanagan, Arlette C. Perry

**Affiliations:** Department of Kinesiology and Sport Sciences, University of Miami, Coral Gables, FL 33146, USA; e.white6@umiami.edu

**Keywords:** childhood obesity, physical inactivity, body image, physical literacy, minority health, women’s health

## Abstract

Body Dissatisfaction (BD) and low physical self-concept and exercise efficacy have been linked to poor physical fitness levels and adverse health outcomes in children. The purpose of this study was to examine the relationship between BD, physical fitness, exercise self-efficacy, and self-Perception of Physical Fitness (PFP) in Latina and Black female children. Twenty-eight Latina and Black children enrolled in an elementary afterschool program, aged 8–12, completed surveys evaluating body dissatisfaction, exercise efficacy, PFP, and measures of physical fitness. Subjects exhibited moderate but significant inverse relationships between BD and PFP in strength (*r* = −0.459), agility (*r* = −0.382), aerobic fitness (*r* = −0.354), and flexibility (*r* = −0.461) (*p* < 0.05 for all). There was a significant negative correlation between exercise efficacy and BD (*r* = −4.2; *p* < 0.05). Power (*r* = 0.51) and flexibility (*r* = 0.42) were the only physical fitness measures significantly and positively related to children’s PFP (*p* < 0.05). A significant medium inverse relationship was also found between BD and aerobic fitness scores (*r* = −0.381; *p* < 0.05). However, after controlling for exercise efficacy or perception of physical fitness, the relationship between BD and aerobic fitness was not significant (*p* > 0.05). Findings suggest that positive PFP and positive performance in several physical fitness measures are associated with lower levels of BD in minority female children. Furthermore, evidence suggests exercise efficacy and PFP can mediate the relationship body image and aerobic fitness. These findings suggest that PFP, more so than measured physical fitness, was associated with lower levels of BD in minority female children. These results have important implications for programs designed to improve physical fitness and mental health in minority children.

## 1. Introduction

Given the current global obesity epidemic, Physical Activity (PA) is currently used as a common intervention for weight loss and weight maintenance in youth [1]. Levels of PA are low and sedentary behaviors are remarkably high in children and adolescents, with females being less active than males [2,3]. On average, only 36% of female youth aged 6–11 meet national PA guidelines, and this number drops drastically to only 3% after the age of 12 [2] with Black and Latina girls, exhibiting even lower rates of PA participation than their non-Latino White peers [2,4]. The age range of 10–15 years is considered one of the most critical periods of childhood maturation and the development of overweight and obesity in adolescents [5]. The World Health Organization now identifies physical inactivity as the fourth leading risk factor for global mortality, causing an estimated 3.2 million deaths globally [6]. Poor nutrition coupled with low PA levels can lead to obesity and its associated co-morbidities, including type 2 diabetes and hypertension [7], both of which are more prevalent among Latino and Black populations [8,9]. Obesity rates are highest in minority children, with 22.4% of Latino children and 20.2% of Black children reported as being obese [10].

Negative mental health may be a consequence of weight gain and physical inactivity. Overweight and obese individuals often express a higher level of dissatisfaction with their bodies and a more negative body image than their normal weight peers [11]. Body Dissatisfaction (BD) refers to the negative subjective evaluations of one’s physical body, such as figure, weight, stomach, and hips [12]. Unfortunately, BD is alarmingly high in children and adolescents, particularly in females. Maloney et al. reported that 55% of third-sixth grade girls expressed desires to be thinner [13], while Rolland et al. [14] found that 50% of eight to thirteen year olds reported concerns about their body weight with 40% of those children attempting weight loss efforts [15]. Children and adolescents who are concerned with their weight are also more likely to develop negative eating behaviors and attitudes such as bingeing, purging, fasting, and the development of clinical eating disorders [16]. Furthermore, a negative body image is often correlated with decreased physical activity and physical fitness levels [17]. Although White girls tend to possess higher levels of BD and body image concerns, BD has been on the rise in Latina and Black girls [18,19,20].

Faigenbaum, et al. [21] recently proposed the pediatric Physical Inactivity Triad (PIT): a myriad of exercise deficit disorder, pediatric dynapenia, and physical illiteracy to call attention to the complex interrelationship of variables that increase risk of childhood obesity. In the PIT model, physical illiteracy is defined as the lack of confidence, competence, motivation, and knowledge to move proficiently in a variety of physical activities. According to this model, physical illiteracy will lead to a lack of motivation, participation, and engagement of physical activities ultimately resulting in reduced physical activity participation (exercise deficit disorder) and muscle weakness (pediatric dynapenia). It is our opinion that this model may help to explain the low physical fitness levels and sedentary behaviors observed among children globally today [2,3,22].

Physical Fitness Perception (PFP) describes one’s perception of sport competence and physical fitness abilities, such as strength and endurance. Research suggests that a positive physical self-concept is positively correlated to physical activity levels [23] and that athletic components of self-concept are significantly and positively associated with body image [24]. Therefore, PFP may be an important contributor to the relationship between negative body image and physical fitness in minority female youth. The present study was done to investigate whether BD is associated with PFP in minority females, and if PFP and exercise efficacy can be a mediator of the relationship between BD and physical fitness, in Latina and Black girls.

## 2. Methods

A total of 28 female minority elementary-school children aged eight to twelve years (15 Black, 13 Latina) from four different Miami-Dade County afterschool programs volunteered to be in this study and completed all necessary assessments and testing. The participating schools were students of predominately Latino or African-American descent. Although no information was provided on Socio-Economic Status (SES) of participants, most qualified for free or reduced-rate lunches, indicating the majority of students were of lower SES. All four schools had partnerships with the YMCA of South Florida with whom we conducted our research. Racial demographics were provided by the YMCA. All procedures were approved by the Institutional Review Board for use of human subjects on 09/30/2016, HSRO No. 20160719. Parents completed signed informed consent forms, and all children completed assent forms before participating in the study.

### 2.1. Physical Measures

On the first day of testing, all subjects were evaluated for height, weight, blood pressure, and heart rate. Height (cm) and weight (kg) were measured using a wall-mounted stadiometer and digital scale. Body Mass Index (BMI) was computed using kg/m^2^.

### 2.2. Body Image Satisfaction

The Collins Body Figure Perception survey [25] was used to determine BD, by calculating the difference between what participants considered to be an ideal shape compared with what they considered to be their actual body shape [2]. The body dissatisfaction scores ranged from zero to six and have a reported three-day test-retest reliability of 0.71 [26]. The greater the difference between perceived body shape and actual body shape, regardless of the direction, the greater the BD score.

### 2.3. Physical Fitness Perception and Exercise Efficacy

The children’s’ PFP was evaluated to determine the performance perception of various tests of physical fitness (muscular strength, aerobic fitness, power, muscular endurance, flexibility, and agility). Each of the physical fitness categories had been previously taught to familiarize students with the terminology. Examples of tests for the five physical fitness categories were provided. The child’s perceptions were scored using a Likert scale with 1 being the lowest and 5 being the highest perception of performance. The numbers also corresponded to the following phrases: poor (1), below average (2), average (3), above average (4), and excellent (5). A modified version of this questionnaire has been used in the Health Behavior in School-Aged Children study [27] and in a study by Gestsdottir et al. [28] evaluating aerobic fitness. Exercise self-efficacy and confidence was evaluated using an exercise self-efficacy scale which required participants to rate how well they believed they could overcome obstacles related to exercise performance and physical activity [29]. Each of the 10 questions was answered using a 10-point Likert scale, with one being the lowest confidence rating and 10 being the highest.

### 2.4. Physical Fitness

On a separate day, five physical fitness tests were performed: upper body strength, aerobic fitness, lower body power, muscular endurance, flexibility, and agility. Hand Grip Strength (HGS) was used as a surrogate measure of upper body strength [30]. Subjects were required to maximally squeeze a Jamar hydraulic hand held dynamometer using the dominant hand (J.A. Preston Corporation, Clifton, NJ, USA) according to procedures set forth by the American Society of Hand Therapists [31]. Participants performed the test twice on the dominant arm and the highest value achieved in kg was recorded. Aerobic fitness was assessed using the Two-Minute Walk Test (2MWT) [32]. Subjects were instructed to walk a 30.5-m course (15.24 m in each direction) as quickly as possible with 180 degree turns at each end [33]. The maximum distance covered in two minutes was recorded. Lower body power was measured via the Vertec Jump Training System (Sports Imports, Hilliard, OH, USA). The vertical jump was calculated by subtracting a subject’s standing reach height from her maximal jump height [34]. Abdominal muscular endurance was measured via an abdominal curl up test according to procedures set forth from The President’s Challenge Physical Activity and Fitness Awards Program [35]. The maximum number of curl ups that the child could perform in one minute while resting briefly as needed to complete the test was recorded. Flexibility of the lower back and hamstrings was assessed using a sit and reach box (Acuflex I, Novel Products, Inc., Rockton, IL, USA). The Sit and Reach Test (SRT) was conducted using standard procedures, as described by Butler et al. [36]. The maximum distance a subject could extend her arms and reach forward while knees were extended with feet abutting the box was recorded in cm. Agility was measured using a shuttle run performed in accordance with the standard guidelines from the Cooper Institute Fitnessgram [37]. Two parallel lines were marked 9.14 m apart from one another and 2 foam blocks (5.08 cm × 5.08 cm × 10.16 cm) were placed behind one of the lines. Subjects ran back and forth as quickly as possible to line up two foam blocks on the opposite line while picking up one foam block at a time. In total, two shuttles were completed. Scores were recorded to the nearest tenth of a second.

### 2.5. Statistical Analysis 

All data were analyzed using correlation statistics in an SPSS statistical package (version 24, IBM SPSS Inc., Armonk, NY, USA). Means (M) and Standard Errors (SE) were calculated for all physical variables, BD, PFP, and actual physical fitness. Data were initially screened for normality by calculating skewness and kurtosis; values between ±2.00 were deemed normal [38]. Pearson’s correlations were then performed using a correlation matrix to examine relationships between body image, physical fitness measures, and PFP. Significance was accepted at *p* ≤ 0.05. Effects of correlations were interpreted using Cohen, 1988 [39].

## 3. Results 

Shown in Table 1 are the physical characteristics and BD scores of Latina and Black children. The average BMI of participants was 20.78, which is within the average range. Shown in Table 2 are the perceived and actual physical fitness scores. Not reported in table form, is the non-significant relationship between BMI and BD (*p* > 0.05).

Perceived flexibility performance was positively and significantly correlated with measured flexibility performance (*r* = 0.42, *p* ≤ 0.05), and perceived power was positively and significantly correlated with actual lower body power (*r* = 0.51, *p* ≤ 0.01). There were no other significant correlations between perceived and actual physical fitness measures found in our sample of children (Table 3).

There was a moderate, significant negative correlation between BD and perception of flexibility (*r* = −0.46, *p* ≤ 0.01), perception of grip strength (*r* = −0.46, *p* ≤ 0.01), perception of agility (*r* = −0.38, *p* ≤ 0.05), and perception aerobic fitness (*r* = −0.35, *p* ≤ 0.05) (Table 3). The higher the BD, the lower the child’s perceived performance on each of the aforementioned fitness variables. There were no other significant correlations observed between BD and perceived physical fitness scores. The average exercise efficacy score was 77.56 ± 2.99. There was a negative significant correlation between exercise efficacy and BD (*r* = −0.42, *p* ≤ 0.05). Thus, the greater the BD, the lower the exercise self-efficacy. Exercise self-efficacy scores were also positively correlated with measured lower-body power (*r* = 0.48, *p* ≤ 0.05) and perception of strength (*r* = 0.43, *p* ≤ 0.05). Aerobic fitness was the only measured physical fitness variable that was significantly and inversely related to BD (*r* = −0.34, *p* ≤ 0.05) (Table 4). This indicates that the greater the BD, the lower the actual aerobic fitness score. However, after controlling for perception of aerobic fitness or exercise self-efficacy, this relationship failed to maintain significance.

When examining results by race, the significant aforementioned relationships were maintained between BD and perceptions of strength (*r* = −0.56, *p* ≤ 0.05), agility (*r* = −0.58, *p* ≤ 0.05), and flexibility (*r* = −0.48, *p* ≤ 0.05) concomitant with BD and perception of power (*r* = −0.53, *p* ≤ 0.05) in Black girls only. In Latina girls however, only a positive and significant relationship between BD and perception of power (*r* = 0.73, *p* ≤ 0.01) was found.

## 4. Discussion

In general, PFP and self-exercise efficacy in Latina and Black girls were related to feelings of body satisfaction. This indicates that perception of children’s fitness and their exercise self-efficacy contribute to physical confidence, physical fitness abilities, and sport competence, all of which encompass the physical literacy component of the PIT model [21]. Given the significant association between PFP and exercise self-efficacy with body image in our study and in the literature [24,40], these findings may serve to influence physical activity participation and ultimately physical health in youth, as proposed by Faigenbaum [21].

Our sample of minority children evidenced BMI levels (20.78 ± 0.77), which were well below the 85 and 95th percentile cut-points [41,42] for overweight and obesity supporting average BMI levels for our sample of minority children. Although BMI levels fell within the average range, 50% of participants were dissatisfied with their bodies selecting ideal figures different than self-perceived figures. Latina girls demonstrated a higher occurrence of BD at 60% compared to their Black peers at 41.2%. The tendency of Latino children to show higher rates of BD than their White peers has been previously reported [43]. The correlation between BD and BMI was not significant in our sample of Latina and Black children. This is in contrast to other studies, which show that BD was significantly related to BMI in White, Latina, and Black children, with higher BMI levels being associated with higher BD [14,44]. Therefore, other social or cultural issues unrelated to body fat or body weight, may have influenced BD in our sample.

In adults, research tends to show a significant correlation between physical fitness, physical activity levels, and mental health scores [45,46,47]. Investigators have concluded that being physically fit has a profound positive effect on mental health. However, in young individuals information on this relationship is lacking [48] and to our knowledge, no information on this relationship has been reported in minority children. In one of the few studies examining this relationship in youth, Gestsdottir et al. found that body image was positively correlated with the perception of one’s aerobic fitness in 15-year-old youth, indicating that the higher the perception of aerobic fitness, the lower the BD [28]. Although perception of aerobic fitness and body image in adolescents in that study were positively related, actual aerobic fitness was a stronger correlate of body image. However, that study was done in predominately white Icelandic youth with a subject population older than children in the current study. In our study, PFP had a stronger relationship with BD than measured fitness in several physical fitness components.

In our sample of minority children, the perception of flexibility, strength, agility, and aerobic fitness were all moderately correlated with BD [39]. Thus, the stronger, more flexible, and aerobically fit the child felt, the lower their negative body image perceptions. However, upon further analyses, we found these results were driven by the strong relationship between perception of physical fitness and BD in Black girls. In four of the six fitness variables, perceptions of strength, agility, flexibility, and power were significantly related to BD. In contrast, in Latina girls, the higher the perception of power performance, the lower their body satisfaction. Thus, although it appears that perceptions of agility, flexibility, and strength were related to a better body image, this was the case primarily for Black participants. Although BD is reported to be greater in Latina than in Black girls, their PFP does not appear to be linked to levels of body satisfaction. Given the limited sample size, more information on Latina and Black girls is needed. This has important implications for future research emphasizing physical activity programs for minority youth, since improvements in one’s PFP may be significantly associated with body image. Given the fact that physical activity levels decline dramatically after age 11 and are significantly lower in Latina and Black than White children [2], this highlights the need for programs embracing physical activity promotion and exercise efficacy as a way to improve both physical and mental health. Our findings are also in agreement with previous reports stating that children who feel good about their physical fitness also tend to feel good about their appearance [28,49].

Few studies have examined all components of physical fitness in relation to the child’s perception of all components of physical fitness. Of the six components of physical fitness measured, only two (power and flexibility) were correlated with children’s perception of their performance in these skills. This indicates that in general, minority children have an inaccurate self-concept of their physical fitness at this age, or do not fully comprehend the skills required of each of the physical fitness components. It would be interesting to examine all components of physical fitness, in order to determine if race differences do exist with regard to perception and actual physical fitness levels and moving forward, whether this relationship tracks into adolescence and adulthood. Given the fact that PFP and body satisfaction were significantly related across a number of fitness components, enhancing fitness perception and confidence may be an important factor to consider when designing physical activity programs for children [21].

Aerobic fitness was the only measured physical fitness variable significantly correlated to body image. However, after controlling for either perception of aerobic fitness or exercise self-efficacy, the relationship failed to reach statistical significance. This again indicates that perception of aerobic fitness may be more closely related to body satisfaction.

## 5. Limitations

It is important to note there were several limitations to the study. Since Latino and Black children appeared to have different levels of self-concept and evidenced different correlations between PFP and BD, more research needs to be done using a larger sample size. Although many correlations were significant, they were moderate. This reinforces the need to extend this study to examine a larger number of minority children while also including both girls and boys.

## 6. Conclusions

In the present study, BMI was not associated with BD indicating that, perhaps, factors other than BMI may influence BD in our sample of minority female children. Additionally, the perception of several measures of physical fitness were significantly related to BD in our sample; however, these results were driven by the strong relationships between PFP and BD in Black girls. Future studies need to extend these findings using a larger sample size to clarify whether relationships between PFP and BD differs by race in minority children. Aerobic fitness was the only measured fitness variable to be related to BD. Therefore, programs aimed at improving exercise perception and efficacy as noted by Faigenbaum [21] may be an optimal approach to increasing physical activity levels, enhancing body image, and physical health in female youth of all racial groups. Given that physical activity levels are lower in Latina and Black female children compared to their White peers [2,4], these findings may have important implications for exercise programming and obesity prevention in minority children.

## Figures and Tables

**Table 1:** Physical Characteristics, BD, and Exercise Self-Efficacy Scores of Latina and Black Children (Mean ± SE).

Variable	Mean (*n* = 28)
Age	9.79 ± 0.20
Height (cm)	143.97 ± 1.62
Weight (kg)	43.13 ± 2.00
Body Mass Index	20.78 ± 0.77
Body Dissatisfaction	0.58 ± 1.78
Exercise Self-Efficacy	77.56 ± 2.99

Note: SE is standard error.

**Table 2:** Perception of Physical Fitness and Measured Physical Fitness Outcomes of Latina and Black Children (Mean ± SE).

Variable	Perception of Physical Fitness Mean (*n* = 28)	Actual Physical Fitness Mean (*n* = 28)
Muscular Endurance	3.38 ± 0.81	38.80 ± 1.99
Strength	3.56 ± 1.96	8.76 ± 0.55
Power	3.66 ± 0.23	30.15 ± 1.52
Agility	3.97 ± 0.19	13.14 ± 0.25
Aerobic Fitness	3.51± 0.20	228.87 ± 3.95
Flexibility	3.78 ± 0.21	30.39 ± 1.20

Note: SE is standard error. Perception was based on how well participants thought they would perform on each physical fitness measure, using a Likert scale, with 1 being the lowest and 5 being the highest score. Physical fitness outcomes were reported as follows: endurance reported curl-ups completed in one minute (raw number), strength reported as a grip dynamometer (kg), power reported as jump height (cm), agility reported as time to complete a shuttle run (s), aerobic fitness reported as distance covered in two-minute walk test (m), and flexibility reported as sit-and-reach score (cm).

**Table 3:** Pearson correlations between BD and Perception of Physical Fitness and Measured Physical Fitness Scores.

Variable	Perception of Physical Fitness Correlation Coefficient (*r*)	Actual Physical Fitness Correlation Coefficient (*r*)
Muscular Endurance	−0.276	0.053
Strength	−0.459 *	−0.225
Power	−0.302	−0.274
Agility	−0.382 *	0.169
Aerobic Fitness	−0.354 *	−0.381 *
Flexibility	−0.461 *	−0.013

Note: Correlation coefficients were determined using Pearson’s correlation coefficient. * indicates significant correlations, *p* < 0.05.

**Table 4:** Pearson correlations (*r*) between Perception of Physical fitness and Children’s Measured Physical Fitness Values.

Variable	Correlation Coefficient (*r*)
Muscular Endurance	0.053
Strength	0.109
Power	0.514 **
Agility	−0.169
Aerobic Fitness	0.206
Flexibility	0.421 *

Note: Correlation coefficients were determined using Pearson’s correlation coefficient. * indicates significant correlations, *p* < 0.05. ** indicates significant correlations *p* < 0.005.
